# Optimization of a DiCre recombinase system with reduced leakage for conditional genome editing of *Cryptosporidium*

**DOI:** 10.1186/s13071-024-06431-1

**Published:** 2024-08-21

**Authors:** Yue Huang, Jinli Li, Shifeng Pei, Heng You, Huimin Liu, Yaqiong Guo, Rui Xu, Na Li, Yaoyu Feng, Lihua Xiao

**Affiliations:** 1 State Key Laboratory for Animal Disease Control and Prevention, South China Agricultural University, Guangzhou, 510642 China; 2 Guangdong Laboratory for Lingnan Modern Agriculture, Center for Emerging and Zoonotic Diseases, College of Veterinary Medicine, South China Agricultural University, Guangzhou, 510642 China

**Keywords:** *Cryptosporidium*, DiCre, Conditional gene knockout, Leaky activity, Promoter

## Abstract

**Background:**

The dimerizable Cre recombinase system (DiCre) exhibits increased leaky activity in *Cryptosporidium*, leading to unintended gene editing in the absence of induction. Therefore, optimization of the current DiCre technique is necessary for functional studies of essential *Cryptosporidium* genes.

**Methods:**

Based on the results of transcriptomic analysis of *Cryptosporidium parvum* stages, seven promoters with different transcriptional capabilities were screened to drive the expression of Cre fragments (FKBP-Cre59 and FRB-Cre60). Transient transfection was performed to assess the effect of promoter strength on leakage activity. In vitro and in vivo experiments were performed to evaluate the leaky activity and cleavage efficiency of the optimized DiCre system by polymerase chain reaction (PCR), nanoluciferase, and fluorescence analyses.

**Results:**

The use of promoters with lower transcriptional activity, such as pcgd6_4110 and pcgd3_260, as opposed to strong promoters such as p*Actin*, p*α-Tubulin*, and p*Enolase*, reduced the leakage rate of the system from 35–75% to nearly undetectable levels, as verified by transient transfection. Subsequent in vitro and in vivo experiments using stable lines further demonstrated that the optimized DiCre system had no detectable leaky activity. The system achieved 71% cleavage efficiency in vitro. In mice, a single dose of the inducer resulted in a 10% conditional gene knockout and fluorescent protein expression in oocysts. These fluorescently tagged transgenic oocysts could be enriched by flow sorting for further infection studies.

**Conclusions:**

A DiCre conditional gene knockout system for *Cryptosporidium* with good cleavage efficiency and reduced leaky activity has been successfully established.

**Graphical Abstract:**

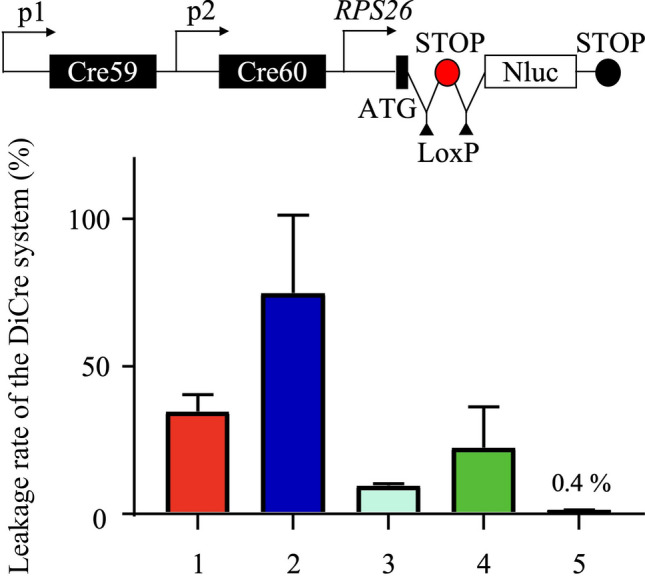

**Supplementary Information:**

The online version contains supplementary material available at 10.1186/s13071-024-06431-1.

## Background

*Cryptosporidium* spp. are common zoonotic parasites that primarily infect the intestinal and gastric mucosa of vertebrates, including humans, livestock, and pets [1]. They pose a greater risk to newborns and immunocompromised individuals, often causing severe and potentially fatal diarrhea [2]. Given the significant threat that *Cryptosporidium* poses to humans and animals, understanding the function of essential genes throughout its life cycle is critical to the identification of potential drug and vaccine targets [3].

To study essential genes in *Cryptosporidium*, it is necessary to establish a conditional gene knockout system. Although the clustered regularly interspaced palindromic repeats (CRISPR)/CRISPR-associated protein 9 (CRISPR/Cas9) technology provides a highly efficient approach to genetic manipulation, it is not a comprehensive solution for knocking out essential genes. To overcome this limitation, specific CRISPR/Cas9-based toolkits for *Cryptosporidium* have been developed, including the destabilization domain of the *Escherichia coli* dihydrofolate reductase (DHFR) system [4, 5], the auxin-inducible degron system [6], and the dimerizable Cre recombinase (DiCre) system [7]. In particular, the DiCre system is characterized by efficient genome editing, complete gene knockout, and adaptability for in vivo studies [8], making it a preferred method for conditional knockout of indispensable *Cryptosporidium* genes.

The application of the DiCre system, based on the bacteriophage Cre recombinase, is challenging in *Cryptosporidium* due to the high leaky activity [7, 9]. This system works by independently expressing two inactive fragments of the Cre enzyme (Cre59 and Cre60), each linked to a different rapamycin (RAPA)-binding protein (FKBP12 and FRB). Administration of RAPA triggers heterodimerization of these components, reactivating the recombinase to precisely excise DNA sequences flanked by LoxP sites [10, 11]. While successfully implemented in various apicomplexan parasites, including *Cryptosporidium*, for studying the function of essential genes [7, 9, 12–16], its application in *Cryptosporidium* faces notable limitations. In particular, target genes are often prematurely knocked out in the absence of RAPA (leaky activity), leading to misinterpretation of gene function at specific *Cryptosporidium* developmental stages [7, 9]. Therefore, further optimization and refinement of the DiCre system is essential for accurate research results.

The selection of appropriate promoters for expression of Cre fragments may address high leaky activity [11]. In *Cryptosporidium*, several promoters are used, including p*Actin* for Cas9 [17], p*Enolase* for the resistance gene [18], and p*α-Tubulin* for Cre fragment genes [7]. These housekeeping gene promoters ensure high levels of expression throughout the life cycle. However, such high expression can lead to the accumulation of Cre fragments in the nucleus, causing non-specific interactions and high leaky activity [11]. Using promoters with lower transcriptional activity may reduce nuclear accumulation and mitigate this problem [11].

Here, we present a modified DiCre conditional gene knockout system for *Cryptosporidium* that exhibits remarkably low leaky activity. This improvement uses promoters with reduced transcriptional activity (specifically from cgd6_4110 and cgd3_260) to drive the expression of FKBP-Cre59 and FRB-Cre60. The low background activity was further validated using the HCT-8 cell culture and C57BL/6J mouse models. In addition, the in vivo and in vitro cleavage efficiency of the optimized system was evaluated using quantitative polymerase chain reaction (PCR), luciferase, and fluorescence assays.

## Methods

### Mice, parasite isolates, and cell line

C57BL/6J (C57) and interferon gamma (IFN-γ) knockout (GKO) mice were used in this study. They were obtained from the Guangdong Medical Laboratory Animal Center (Guangzhou, China) and the Jackson Laboratory (Bar Harbor, ME, USA), respectively. For each experiment, mice were matched for sex and age, and were used at the age of 3–4 weeks.

The *Cryptosporidium parvum* IIdA20G1-HLJ isolate was originally obtained from a dairy calf during a cryptosporidiosis outbreak [19]. This isolate was maintained and propagated in the laboratory by sequential passages in GKO mice as described previously [20]. The species and subtype identity of the isolate were confirmed by sequence analysis of the small subunit (SSU) rRNA and 60 kDa glycoprotein (GP60) genes. Oocysts were purified from the feces of infected mice using discontinuous sucrose and cesium chloride gradients as described previously [21].

Human ileocecal colorectal adenocarcinoma (HCT-8) cells (ATCC CCL-244) were obtained from the Shanghai Branch of the Chinese Academy of Sciences. They were cultured in RPMI 1640 medium containing 10% fetal bovine serum (FBS) and 1% penicillin–streptomycin solution, and maintained at 37 °C in 5% CO_2_.

### Transcriptome analysis

Based on the transcriptome analysis of sporozoites and HCT-8 cells infected with *C. parvum* IIdA20G1-HLJ isolate (BioProject no. PRJNA1011005), we selected promoters with distinct transcriptional activity driving gene expression throughout the life cycle: p*α-Tubulin* (cgd4_2860), p*Actin* (cgd5_3160), and p*Enolase* (cgd5_1960) exhibited high transcriptional activity, and p*E2F* (cgd1_1570) and p*ATPase* (cgd2_1360) showed moderate transcriptional activity, while pcgd6_4110 and pcgd3_260 displayed the weakest transcriptional activity. Their transcriptional capacity was evaluated using fragments per kilobase of transcript per million mapped reads (FPKM), calculated with RSEM software (v1.3.1, https://github.com/deweylab/RSEM.git), and the gene expression levels were visualized as line chart using the R package ggplot2.

### Plasmid construction for *C. parvum* sporozoites transiently transfected

**Fig. 1:**
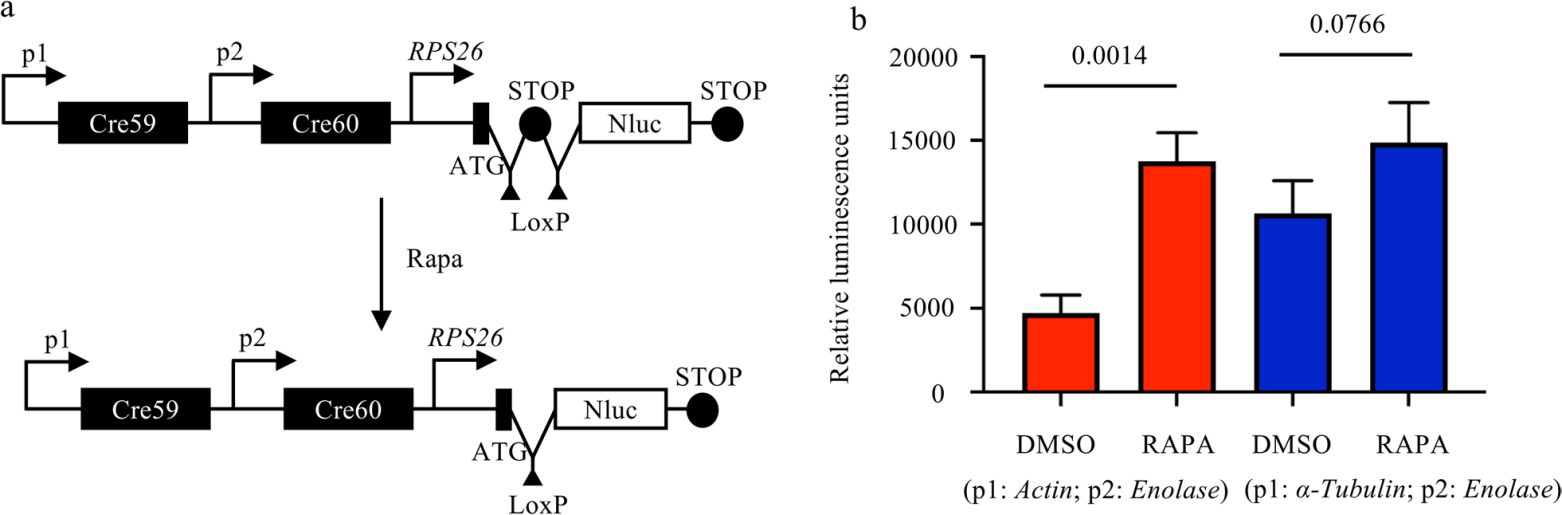
Analysis of the leaky activity in the dimerizable Cre recombinase (DiCre) system in *Cryptosporidium*. **a** Illustration of the plasmid construct used in the transient Cre recombinase assay. Two different promoters (p1 and p2) were used to drive the expression of the inactive Cre59 and Cre60 fragments of the Cre enzyme, each linked to the rapamycin-binding protein, FKBP12 or FRB. Upon induction by rapamycin, the two fragments combine to form an active Cre enzyme that excises a floxed stop sequence, thereby activating the expression of Nluc. **b** Comparison of luciferase activity in HCT-8 cell cultures infected with *Cryptosporidium* that had been transiently transfected with the indicated construct for 24 h with or without rapamycin (*P* = 0.014 for *Actin* and *Enolase* promoters, *P* = 0.0766 for *α-Tubulin* and *Enolase* promoters). Luciferase expression was observed in the absence of an inducer when *Actin* and *Enolase* or *α-Tubulin* and *Enolase* promoters were used to drive the expression of the Cre fragments, suggesting the presence of leakage activity in the system

The nucleotide sequences of wild-type FKBP-Cre59 and FRB-Cre60 were optimized for the *C. parvum* codon and synthesized by GENEWIZ (Waltham, MA, USA) (Additional file 4: Table S1). The plasmid pUC19 was used as the base plasmid for the assembly of key elements to evaluate the functionality of the DiCre system. First, a fragment containing the promoters (Additional file 5: Table S2) identified from the transcriptome analysis and driving Cre59-FKBP and Cre60-FRB sequences was inserted into the pUC19 plasmid. Next, a fragment containing the 40S ribosomal protein S26 (RPS26) promoter (Additional file 5: Table S2) driving a nanoluciferase (Nluc) sequence was inserted into the pUC19 plasmid. Finally, the 3′ untranslated region (UTR) region of RPS26, flanked by LoxP sites, was inserted between the p*RPS26* and the Nluc coding sequence (Fig. 1a).

We randomly combined different promoters to drive the expression of Cre59-FKBP and Cre60-FRB. The following plasmids were constructed: pStopLoxP-Nluc-Act/Eno, pStopLoxP-Nluc-Tub/Eno, pStopLoxP-Nluc-E2F/ATPase, pStopLoxP-Nluc-cgd3_260/ATPase, and pStopLoxP-Nluc-cgd6_4110/cgd3_260.

### Construction of *C. parvum* mutants

To evaluate the leakage activity of the optimized DiCre system, a StopLoxP-Nluc line was constructed using a guide RNA (gRNA) targeting the 3′ end of the *TK* gene. The sequences flanking the *TK* gene were used as homologous arms to insert the DiCre, neo, and LoxP-3′ UTR-LoxP-Nluc cassettes (Additional file 1: Fig. S1a). Similarly, another Nluc-neoLoxP-mNG line was constructed by inserting the DiCre and LoxP-Nluc-neo-LoxP-mNeonGreen (mNG) cassettes into the *TK* locus (Additional file 1: Fig. S1b).

### Generation of transgenic parasites

Sporozoites were harvested from *C. parvum* oocysts that were excysted using bleach and sodium taurocholate treatment as described previously [22]. A total of 5 × 10^7^ sporozoites were resuspended in SF buffer (Lonza, Basel, Switzerland) containing the donor and CRISPR/Cas9 plasmids (50 µg each in 100 μl). Electroporation was performed using the EH100 program on an AMAXA 4D-Nucleofector System (Lonza, Basel, Switzerland) as described previously [18]. Two GKO mice were given 200 µl of 8% sodium bicarbonate by oral gavage. Five minutes later, each mouse was inoculated with 2.5 × 10^7^ electroporated sporozoites [23]. At 18 h post-infection (HPI), paromomycin was added to the drinking water at a concentration of 16 g/l to select for transgenic parasites.

### Verification of the correct gene integration

PCR analysis was used to verify correct gene integration. The PCR reaction contained 1 µl of DNA extracted from fecal pellets using the Omega Stool DNA Kit (Omega Bio-tek, Norcross, GA, USA), Phanta Max Super-Fidelity DNA Polymerase (Vazyme, Jiangsu, China), and the primers listed in Additional file 6: Table S3. PCR amplification was performed on a Veriti 96-well thermal cycler (Bio-Rad, Hercules, CA, USA). PCR products were identified by electrophoresis using 1.0% agarose gel containing GelRed (1:10,000).

### Assessment of gene expression leakage

To evaluate the minimal leakage of the optimized DiCre system in vitro, HCT-8 cells were cultured in medium with or without 200 nM RAPA (Aladdin, Shanghai, China), a concentration higher than the previously reported 100 nM, to ensure the efficacy of the drug [7, 9], and infected with *C. parvum* sporozoites transiently transfected with the plasmids or a *C. parvum* line stably transfected with StopLoxP-Nluc. Relative luminescence units (RLU) were measured at 24 HPI for the sporozoites transiently transfected with the plasmid, or at 2, 12, 24, 36, and 48 HPI for the StopLoxP-Nluc line as described by Pawlowic et al. [22]. Leakage rate = (RLU_DMSO_ − RLU_Blank_)/(RLU_RAPA_ − RLU_Blank_); DMSO = dimethyl sulfoxide.

For in vivo assessment of leakage, six C57 mice housed separately in individual cages were infected with 10^5^ oocysts of the StopLoxP-Nluc line. On day 4 post-infection (DPI 4), three mice in the experimental group received 200 μg of sirolimus (Huadong Medicine, Hangzhou, China) orally, and three mice in the control group received an equal amount of deionized water (dH_2_O). Fecal samples were collected every 2 days, weighed, and analyzed for luciferase activity.

### Evaluation of cleavage activity

PCR, Nluc, and fluorescence analyses were used to evaluate the in vitro cleavage activity of the optimized DiCre system. HCT-8 cells were cultured in medium with or without 200 nM rapamycin (Aladdin, Shanghai, China), infected with oocysts of the Nluc-neoLoxP-mNG line, and analyzed for cleavage efficiency. The PCR analysis was performed using primers flanking the LoxP sites (Additional file 6: Table S3) on DNA extracted from the culture at 24, 48, and 72 HPI using the QIAamp DNA extraction kit (Qiagen, Düsseldorf, Germany). Nluc activity was measured using cultures infected with the transgenic line at 2, 6, 12, 24, 36, and 48 HPI. Fluorescence was observed at 24 HPI using an Olympus BX53 microscope.

For in vivo evaluation of cleavage, four GKO mice were infected with 10^3^ oocysts of the Nluc-neoLoxP-mNG line. On DPI 9 and 13, the mice were given 200 μg of sirolimus by gavage. Fecal samples were collected on DPI 8, 12, 16, and 20 for oocyst purification. Oocysts expressing mNG were sorted and harvested by flow cytometry (FACSAria III, BD Biosciences, USA).

### Statistical analysis

All statistical analyses were performed using GraphPad Prism software. An unpaired *t*-test was used to compare the means of two groups, with *P*-values ≤ 0.05 considered significant.

## Results

### The transient transfection method validates the leakage activity of the current DiCre system

The FKBP-Cre59 and FRB-Cre60 sequences used in the DiCre system were optimized for *C. parvum* codons. This resulted in a reduction of the guanine and cytosine (GC) content from 55 to 33% for FKBP-Cre59 and from 56 to 35% for FRB-Cre60 (Additional file 2: Fig. S2).

To evaluate the leaky activity of the DiCre system, two plasmids (pStopLoxP-Nluc-Act/Eno and pStopLoxP-Nluc-Tub/Eno) were constructed with FKBP-Cre59 and FRB-Cre60 expression driven by promoters for the *Actin* and *Enolase* or the *α-Tubulin* and *Enolase* genes, respectively. Spontaneous luciferase expression was inhibited by a terminator sequence upstream of the luciferase gene. Upon induction with RAPA, the LoxP-flanked terminator was excised, allowing Nluc expression (Fig. 1a). These plasmids were transiently introduced into *Cryptosporidium* by electroporation. Subsequent analysis showed that HCT-8 cells infected with the transfected *C. parvum* exhibited detectable luciferase activity at 24 HPI, independent of RAPA induction, indicating the leaky activity of the existing DiCre system (Fig. 1b).

### DiCre recombinase with weak promoters shows low leakage in the transient transfection model

**Fig. 2:**
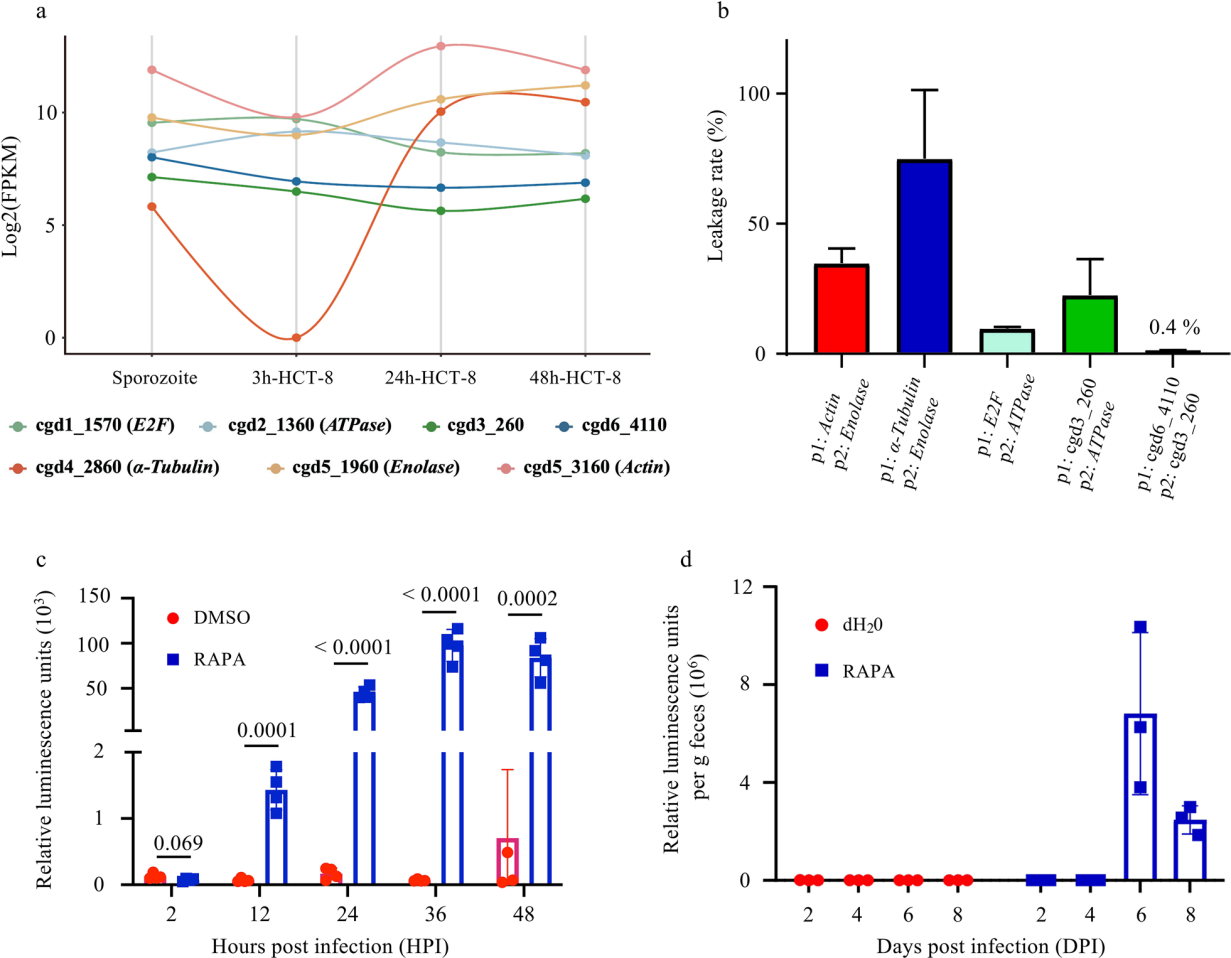
Selection of promoters for reduced leaky activity in the dimerizable Cre recombinase (DiCre) system. **a** Relative expression of genes of interest in sporozoites and developmental stages of *C. parvum* in HCT-8 cells after infection with IIdA20G1-HLJ. Each point represents one gene (*N* = 4). Of particular interest are the genes *Actin*, *Enolase*, *α-Tubulin*, *ATPase*, *E2F*, cgd6_4110, and cgd3_260 in descending order of transcription levels. **b** Leakage rate of the DiCre system using different promoter combinations to drive Cre fragments. Luciferase activity was measured in HCT-8 cell cultures infected with the transient for 24 h with or without rapamycin. The use of cgd6_4110 and cgd3_260 to drive the Cre fragments resulted in the lowest leakage rate. **c** Comparison of luciferase activity in HCT-8 cell cultures infected with the StopLoxP-Nluc line with or without rapamycin induction (*P* = 0.0690, 0.0001, < 0.0001, < 0.0001, and 0.0002 at 2, 12, 24, 36, and 48 HPI, respectively; *N* = 4; bars are standard deviations). **d** Comparison of fecal luciferase activity of C57BL/6J mice infected with the StopLoxP-Nluc line of *C. parvum* with or without rapamycin induction. After the inoculation of mice with StopLoxP-Nluc oocyst, mice in the RAPA group were treated orally with sirolimus on DPI 4, and luciferase activity was detected in the feces on DPI 6 and 8. In contrast, no luciferase activity was detected in control mice that did not receive rapamycin

Based on the comparative analysis of gene expression in transcriptomic data from different developmental stages of *C. parvum*, promoters of seven genes (*Actin*, *Enolase*, *α-Tubulin*, *ATPase*, *E2F*, cgd6_4110, and cgd3_260) were selected to drive the expression of FKBP-Cre59 and FRB-Cre60 (Fig. 2a). Three plasmids (pStopLoxP-Nluc-E2F/ATPase, pStopLoxP-Nluc-cgd3_260/ATPase, and pStopLoxP-Nluc-cgd6_4110/cgd3_260) were constructed to select promoters with minimal leakage activity in the DiCre system, using plasmids pStopLoxP-Nluc-Act/Eno and pStopLoxP-Nluc-Tub/Eno as controls.

The plasmids were introduced by electroporation into sporozoites, which were used to infect HCT-8 cells. At 24 HPI, cells were lysed, and luciferase activity was measured (Additional file 3: Fig. S3). The DiCre system showed a leakage rate of 35–75% when strong promoters were used. However, when promoters with lower transcriptional activity were employed, particularly the cgd6_4110 and cgd3_260 promoters, the leakage rate dropped to 0.4% (Fig. 2b). This suggests that cgd6_4110 and cgd3_260 promoters are optimal for driving FKBP-Cre59 and FRB-Cre60 expression in the DiCre system, with minimal leaky activity.

### Optimized DiCre system has low leakage

The CRISPR/Cas9 system was used to generate the StopLoxP-Nluc line with the integration of the DiCre cassette into the *TK* locus of *C. parvum* (Additional file 1: Fig. S1a). PCR analysis of the 5′ and 3′ ends of the genetically modified locus in *C. parvum* from GKO mice inoculated with the transgenic line yielded PCR products of the expected size (Additional file 1: Fig. S1a).

HCT-8 cells infected with the StopLoxP-Nluc line of *C. parvum* were treated with or without RAPA. At 2, 12, 24, and 48 HPI, cells from both the DMSO and RAPA groups were harvested and analyzed for luciferase activity. The results showed a significant increase in luciferase activity in the RAPA group from 12 to 48 HPI. In contrast, luciferase activity was almost undetectable in the DMSO group (Fig. 2c).

The spontaneous leakage activity of the new promoters was evaluated in C57 mice that were infected with the StopLoxP-Nluc line. On DPI 4, mice in the RAPA group received 200 μg of sirolimus, while the dH_2_O group received an equivalent volume of dH_2_O. The results showed that the dH_2_O group had no detectable fecal luciferase activity after infection with the StopLoxP-Nluc line. In contrast, luciferase activity was detected in fecal samples from the RAPA group on DPI 6 and 8 after RAPA administration (Fig. 2d). This suggests that the optimized DiCre system allows the parasite to complete multiple replications in mice without losing the sequence flanked by LoxP.

### Optimized DiCre system shows inducible cleavage activity in vitro

A Nluc-neoLoxP-mNG line of *C. parvum* was constructed using the same CRISPR/Cas9 approach (Additional file 1: Fig. S1b). PCR analysis of fecal samples from infected mice showed that the Nluc-neoLoxP-mNG and DiCre cassettes was correctly inserted into the *TK* locus (Additional file 1: Fig. S1b).

**Fig. 3:**
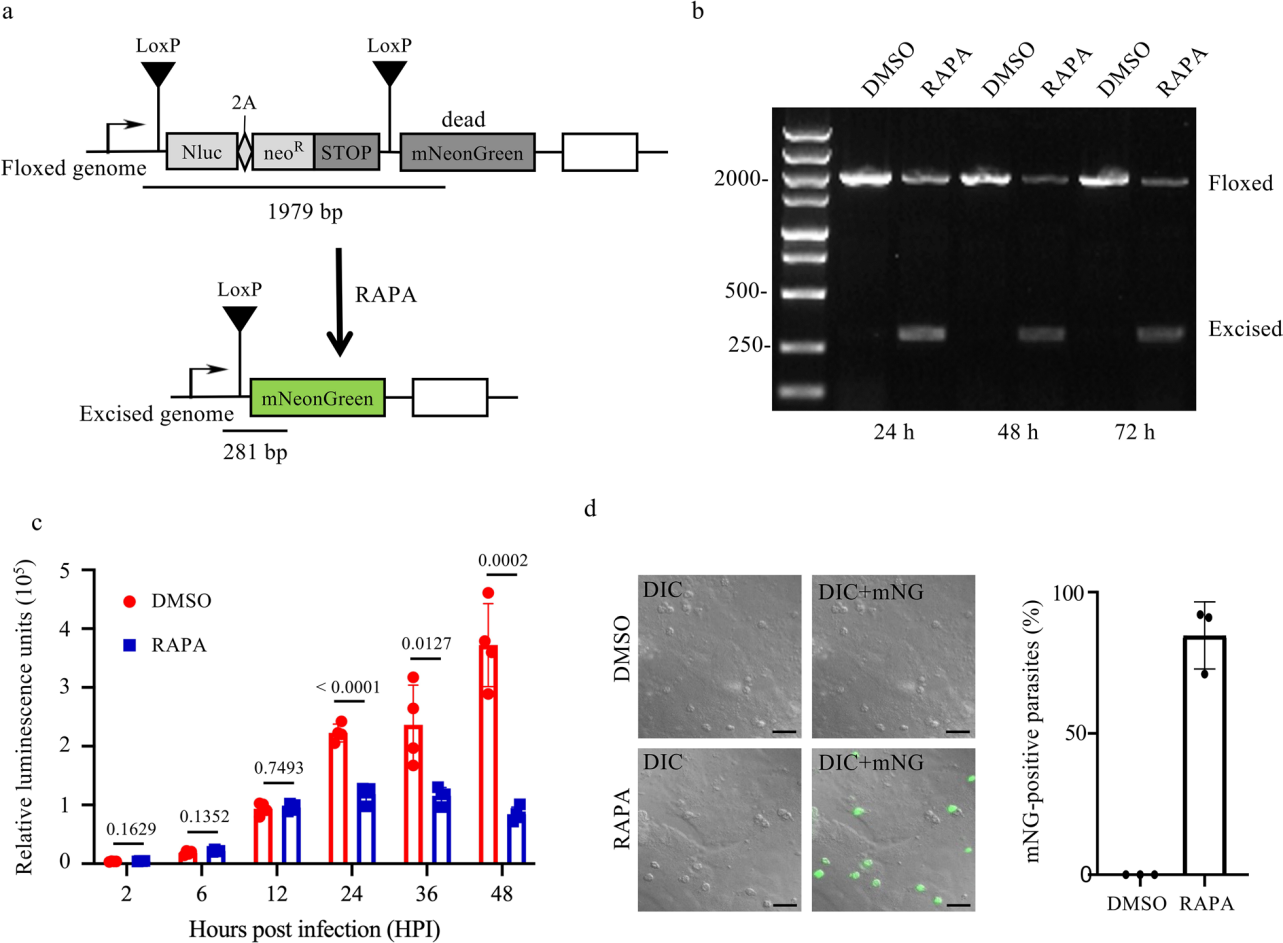
Evaluation of the in vitro cleavage activity of the modified dimerizable Cre recombinase (DiCre) system. **a** Illustration of the principle of Cre-mediated excision of Nluc-neoLoxP-mNG after rapamycin (RAPA) induction. Without cleavage, the PCR-amplified product is 1989 bp in length and the transgenic line has luciferase activity but does not show mNeonGreen (mNG) fluorescence. After enzymatic cleavage by the Cre enzyme, the length of the PCR product is reduced to 281 bp, resulting in the loss of luciferase activity and appearance of mNG fluorescence. **b** PCR evaluation of the cleavage activity of the modified DiCre system. Oocysts of the Nluc-neoLoxP-mNG line were used to infect HCT-8 cell cultures with or without RAPA induction. Genomic DNA was extracted and subjected to PCR analysis. The diagnostic products for the floxed and excised locus are highlighted. RAPA treatment of the cell cultures at 24, 48, and 72 HPI resulted in the generation of excised products. **c** Evaluation of the cleavage activity of the modified DiCre system by a luciferase assay. Infected HCT-8 cell cultures with or without rapamycin induction were analyzed by luciferase assay. Significant differences in luciferase activity were observed (*P* = 0.1629, 0.1352, 0.7493, < 0.0001, 0.0127, and 0.0002 at 2, 6, 12, 24, 36, and 48 HPI, respectively; *N* = 4, bars are standard deviations). **d** Fluorescence assay to evaluate the cleavage activity of the modified DiCre system. Nluc-neoLoxP-mNG-infected HCT-8 cell cultures produced green fluorescent parasites after RAPA induction, which accounted for approximately 71% of the intracellular parasites, compared to no observable green fluorescence in cultures without the RAPA treatment. Scale = 10 μm

HCT-8 cells infected with the Nluc-neoLoxP-mNG line were used to evaluate the cleavage activity of the optimized DiCre system. The PCR analysis using primers flanking the LoxP sites yielded a single band of 1989 base pairs (bp) in the DMSO group. Conversely, with RAPA induction, both the floxed and excised products of 1989 bp and 281 bp were detected at 24, 48, and 72 HPI (Fig. 3a, b). Luciferase activity in the RAPA group plateaued at lower levels at 24 HPI, whereas the luciferase activity in the DMSO group continued to increase (Fig. 3c). Green fluorescence was observed in approximately 71% of the 219 meronts examined at 24 HPI following RAPA induction, whereas no fluorescence was detected in the absence of RAPA induction (Fig. 3d). This indicates that the optimized DiCre system has good cleavage activity in vitro.

**Fig. 4:**
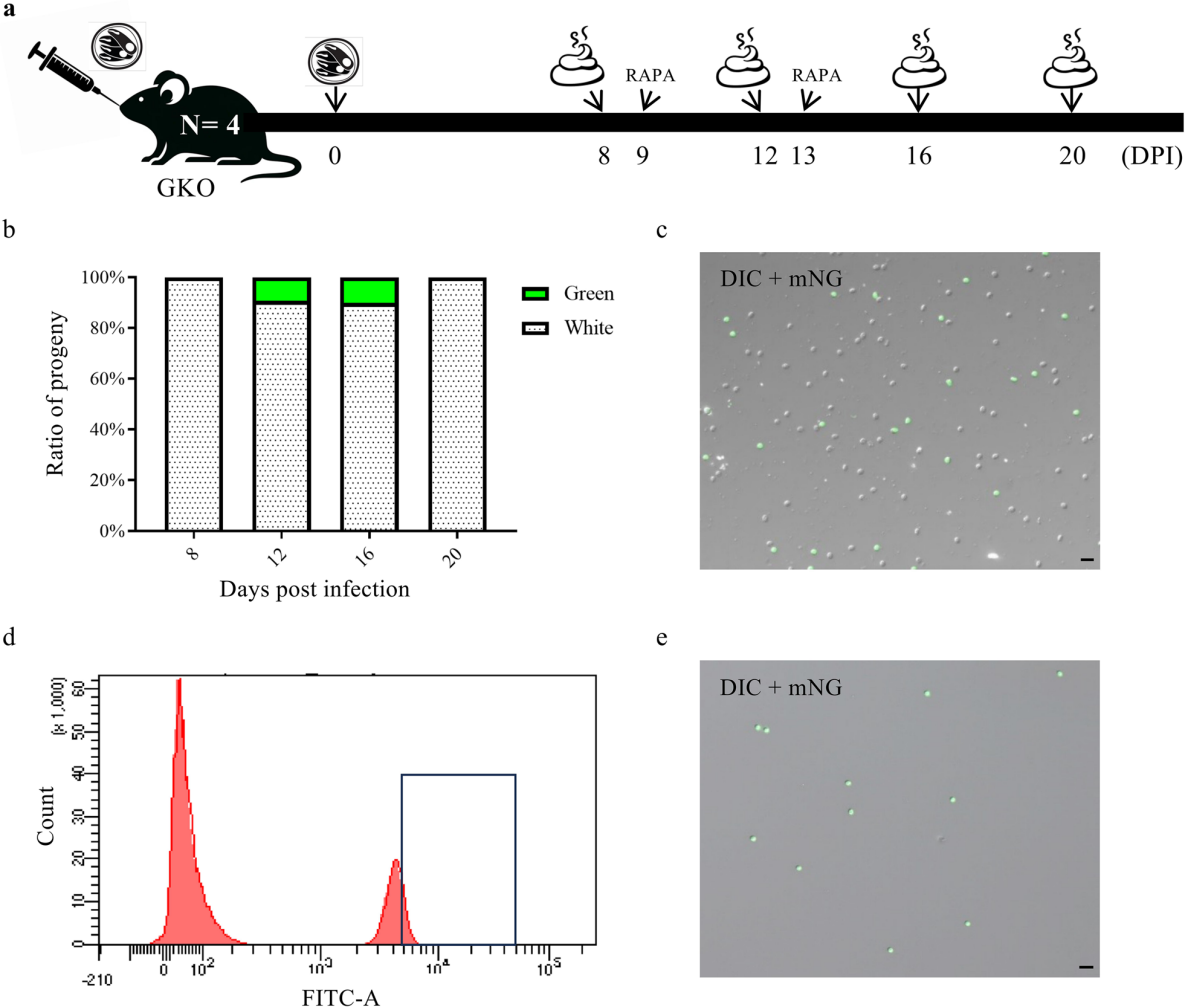
Analysis of the in vivo cleavage activity of the modified dimerizable Cre recombinase (DiCre) system. **a** Infection of GKO mice (*N* = 4) with oocysts of the Nluc-neoLoxP-mNG line, with rapamycin (RAPA) treatment on DPI 9 and 13, and collection of fecal samples for oocyst purification on DPI 8, 12, 16, and 20. **b** Percentage of green oocysts after RAPA induction in samples collected on DPI 8, 12, 16, and 20. **c** Differential interference contrast (DIC) image of oocysts purified from feces on DPI 12 taken under, showing 10– 20% of green oocysts. Scale = 10 μm. **d** Fluorescence intensity distribution in flow cytometric analysis of oocysts purified from the feces on DPI 12. The histogram shows the distribution of cells with fluorescein isothiocyanate (FITC)-A signals, where the horizontal axis represents the intensity of FITC-A signals, and the vertical axis represents number of cells. Oocysts within the selected box were harvested. **e** Image of microscopic analysis of oocysts after flow sorting, showing the presence of only green oocysts. Scale = 10 μm

GKO mice were infected with 10^3^ oocysts of the Nluc-neoLoxP-mNG line of *C. parvum*, followed by treatment with 200 μg sirolimus on DPI 9 and 13. Fecal samples were collected on DPI 8, 12, 16, and 20 for oocyst purification and evaluation of gene excision through the ratio of green oocysts (Fig. 4a).

Approximately 10–20% of the oocysts purified on DPI 12 and 16 showed green fluorescence (Figs. 4b,c). Oocysts were purified from fecal samples and were flow-sorted for green fluorescence (Fig. 4d), which increased the proportion of green oocysts from 10–20% before sorting to nearly 100% after sorting (Fig. 4e).

## Discussion

This report describes the development of a DiCre-based inducible genome deletion system for *C. parvum* characterized by minimal leaky activity. This is achieved by using two promoters (cgd6_4110 and cgd3_260) with lower transcriptional activity than those previously used to drive the expression of two inactive fragments of the Cre enzyme. The leakage of the DiCre system is significantly reduced in both in vitro and in vivo models using both transient and stable lines. Using the Nluc-neoLoxP-mNG line of *C. parvum* constructed with this approach, the modified DiCre system demonstrates an exceptionally high in vitro cleavage efficiency. In GKO mice, a single induction of RAPA resulted in approximately 10% conditional gene knockout in oocysts, which was further enriched by flow cytometry for infection studies. These results suggest that the new DiCre system maintains effective DNA cleavage with reduced leaky activity.

Spontaneous leakage has been associated with conditional gene knockout systems such as the DiCre system, and poses a significant challenge to gene function research. First, in the event that the DiCre conditional system exhibits leaky activity when applied to essential genes, it may lead to the knockout of essential genes located between LoxP sites prior to the RAPA induction, resulting in the death of the target pathogen. Consequently, it would be impossible to select the desired transgenic lines [8]. Second, leakage may result in the premature expression or knockout of genes intended for conditional manipulation at specific developmental stages of the pathogen, thereby leading to the generation of inaccurate and unreliable data in phenotyping studies. Third, when the DiCre system is applied to drug screening, the unintended knockout or expression of target genes may obscure or misrepresent drug effects. This misrepresentation can lead to an underestimation or overestimation of drug efficacy [24].

High leaky activity of the DiCre system is a common problem in studies of protozoan parasites. Data from the present study show that when plasmids containing the DiCre cassette were introduced into *Cryptosporidium*, specific DNA segments were deleted without RAPA induction, indicating high leaky activity. This is consistent with previous observations of the DiCre system in *Cryptosporidium* [7, 9], *Toxoplasma* [16], and *Plasmodium* [12, 25]. However, Hunt et al. [26] and Collins et al. [13] showed low leakage activity in their DiCre systems for *Toxoplasma* and *Plasmodium*. This difference may be due to the different construction of the DiCre expression cassettes, suggesting that although the current *Cryptosporidium* DiCre system is leaky, there is room for optimization.

Results from transient transfection experiments indicate that the expression level of Cre fragments may be a key factor contributing to the leaky activity of the DiCre system. Driving the expression of FKBP-Cre59 and FRB-Cre60 with the weaker cgd6_4110 and cgd3_260 promoters resulted in lower leakage than using potent promoters p*Actin*, p*α-Tubulin*, and p*Enolase*. Previous studies have shown that although the use of promoters with high transcriptional activity increases FKBP-Cre59 and FRB-Cre60 expression in the nucleus, this can lead to the formation of the active Cre complex without induction, resulting in leakage of the DiCre system [11, 16, 25]. Thus, the leakage of the *Cryptosporidium* DiCre system could be reduced by reducing the accumulation of FKBP-Cre59 and FRB-Cre60 in the nucleus through the use of weaker promoters.

The low leakage activity of the system is supported by results of studies conducted with *C. parvum* stably transfected with the modified DiCre cassette. The new *Cryptosporidium* DiCre system showed negligible leakage in both in vivo and in vitro studies, overcoming the problem of the premature cleavage of target genes prior to the RAPA induction [7, 9]. This allows a more accurate assessment of gene function and the effect of gene deletion on *Cryptosporidium* growth and fitness.

The modified DiCre system also shows good cleavage efficiency. Despite the use of promoters with lower transcriptional activity, the system achieved in vitro cleavage efficiency of approximately 70%, which is comparable to the efficiency reported by Tandel et al. [7]. In an infection study, approximately 10% of the oocysts from mice infected with the Nluc-neoLoxP-mNG line showed green fluorescence after a single RAPA induction. The fluorescent pathogens with targeted gene knockouts can be isolated and enriched for infection studies by flow cytometry, which has been used to study functional genes in the related *Toxoplasma gondii* [27]. Furthermore, this approach enables differentiation between essential and non-essential genes: the presence of green fluorescent oocysts after RAPA treatment indicates the dispensable nature of the target genes, while the absence of green oocysts indicates the indispensable nature of these genes.

## Conclusions

In conclusion, the modified DiCre-based inducible genome deletion system for *C. parvum* shows significantly reduced leakage activity and high DNA cleavage efficiency. By using weaker promoters (cgd6_4110 and cgd3_260) to drive Cre fragment expression, spontaneous gene knockout is effectively minimized, ensuring accurate gene function studies. This optimized DiCre system increases the reliability of phenotyping studies and drug screening by reducing unintended gene disruption. Further studies should be conducted using this system to identify critical genes whose deletion results in notable phenotypic changes in *Cryptosporidium*.

### Supplementary Information

Additional file 1. Fig. S1: Illustrations of the *Cryptosporidium *loci modified in this study and confirmation of correct gene integration by PCR analysis. **a** An illustration of the construction of the StopLoxP-Nluc line. The presence of “5′ insert” and “3′ insert” PCR products confirms the correct gene integration. The “TK” product is specific for the *TK* locus. The “control” PCR product is specific for the insulin-like-3 (*INS3*) locus. **b** An illustration of the construction of the Nluc-neoLoxP-mNG line. The presence of “5′ insert” and “3′ insert” PCR products confirms the correct gene integration. The products “Cre,” “Nluc,” and “TK” are specific for the *Cre* (*Cre59* and *Cre60*), *Nluc* and *TK* locus, respectively. The “control” PCR product is specific for the *INS3* locus.

Additional file 2. Fig. S2: Distribution of GC content before and after codon optimization of the FKBP-Cre59 and FRB-Cre60 nucleotide sequences. This optimization reduced the GC content of the FKBP-Cre59 and FRB-Cre60 sequences from 55% and 56% (orange line) to 33% and 35% (blue line), respectively.

Additional file 3. Fig. S3: Comparison of luciferase activity in HCT-8 cell cultures infected with *Cryptosporidium* that had been transiently transfected with the indicated construct for 24 h with or without rapamycin (*P* < 0.001 for *E2F* and* ATPase* promoters, *P* = 0.0405 for cgd3_260 and *ATPase* promoters, *P* < 0.001 for cgd6_4110 and cgd3_260 promoters).

Additional file 4. Table S1: FKBP-Cre59 and FRB-Cre60 sequences optimized for *Cryptosporidium parvum* codon used in this study.

Additional file 5. Table S2: Promoter sequences used in this study.

Additional file 6. Table S3: Primers used in this study.

## Data Availability

All data are included as tables and figures within the article and supplementary information files.
