# Supplementary material for: Optimization of a DiCre recombinase system with reduced leakage for conditional genome editing of Cryptosporidium
Source: Parasit Vectors. 2024 Aug 21;17:352. doi: 10.1186/s13071-024-06431-1 (PMC11337648; doi:10.1186/s13071-024-06431-1)
Supplement: Supplementary file 4 — Additional file 4. Table S1: FKBP-Cre59 and FRB-Cre60 sequences optimized for Cryptosporidium parvum codon used in this study. [file 13071_2024_6431_MOESM4_ESM.docx]

**Table S1.** FKBP-Cre59 and FRB-Cre60 sequences optimized for *Cryptosporidium parvum* codon used in this study

| Name | Sequence (5'→3') |
| --- | --- |
| optimized FKBP-Cre59 sequence | ATGGACTATAAGGACCACGACGGAGACTACAAGGATCATGATATTGATTACAAAGACGATGACGATAAGATGGCCCCAAAGAAGAAGCGGAAGGTCTCTAGAGGTGTTCAAGTTGAAACAATTTCTCCTGGTGATGGTAGAACTTTTCCAAAAAGAGGTCAAACTTGTGTAGTTCATTATACTGGTATGTTAGAAGATGGTAAAAAATTCGACTCATCAAGAGATAGAAACAAACCATTCAAATTCATGTTAGGTAAACAAGAAGTTATTAGAGGTTGGGAAGAAGGTGTTGCTCAAATGTCAGTTGGTCAAAGAGCTAAATTAACTATTTCACCTGATTATGCTTATGGTGCTACTGGTCATCCTGGTATTATTCCACCACATGCTACTTTAGTTTTTGATGTTGAATTATTAAAATTAGAAGCTTCACCATCAAATCCTGGTGCTTCAAATGGATCAACTTCAGACGAAGTTAGGAAAAATTTAATGGATATGTTTAGAGATAGACAAGCTTTTTCAGAACATACTTGGAAAATGTTATTATCAGTTTGTAGATCATGGGCTGCTTGGTGTAAATTAAATTAA |
| optimized FRB-Cre60 sequence | ATGAAAATGGCCCCTAAGAAGAAGAGAAAGGTGTCTAGAATTTTATGGCATGAAATGTGGCATGAAGGTTTAGAAGAAGCTTCAAGATTGTACTTTGGTGAAAGAAATGTTAAAGGTATGTTTGAAGTTTTAGAACCATTACATGCTATGATGGAAAGAGGTCCACAAACTTTAAAAGAAACTTCATTTAATCAAGCTTATGGTAGAGATTTAATGGAAGCTCAAGAATGGTGTAGAAAATATATGAAATCTGGTAATGTAAAAGATTTATTGCAAGCTTGGGATTTGTATTATCACGTTTTTAGAAGAATTTCAGCTTCACCATCAAATCCTGGTGCTTCAAATGGTTCAAATAGAAAATGGTTTCCTGCTGAGCCTGAAGATGTTAGAGATTATTTATTATATTTACAAGCTAGAGGTTTAGCTGTTAAAACTATTCAACAACATTTAGGTCAATTGAACATGTTACATAGAAGATCTGGTTTACCAAGACCATCAGATTCAAATGCTGTTTCATTAGTTATGAGAAGAATTAGAAAAGAAAATGTTGATGCTGGTGAAAGAGCTAAACAAGCTTTAGCATTTGAAAGAACTGATTTCGATCAAGTAAGATCATTAATGGAGAATTCAGATAGATGTCAAGATATTAGAAACTTAGCTTTTTTAGGTATTGCTTATAACACTTTATTAAGAATTGCTGAGATAGCTAGGATTAGAGTTAAAGATATTTCAAGAACTGATGGTGGTAGAATGTTAATTCATATTGGTAGAACTAAAACTTTAGTTTCAACTGCTGGTGTTGAAAAAGCTTTATCATTAGGTGTTACTAAATTAGTTGAAAGATGGATTTCAGTTTCTGGTGTTGCTGATGATCCAAATAATTATTTATTTTGTAGAGTTAGAAAAAATGGTGTTGCTGCTCCATCAGCTACTTCACAATTATCAACTAGAGCTTTAGAAGGTATTTTTGAAGCTACTCATAGATTAATTTATGGTGCTAAAGATGATTCTGGTCAAAGATATTTAGCTTGGTCTGGTCATTCAGCTAGAGTTGGTGCTGCTAGAGATATGGCTAGAGCTGGTGTTTCAATTCCTGAAATTATGCAAGCTGGTGGTTGGACTAATGTTAATATTGTAATGAACTATATTAGGAATTTAGACTCAGAAACTGGTGCTATGGTTAGATTATTAGAAGATGGTGATTAA |
