# Supplementary material for: Optimization of a DiCre recombinase system with reduced leakage for conditional genome editing of Cryptosporidium
Source: Parasit Vectors. 2024 Aug 21;17:352. doi: 10.1186/s13071-024-06431-1 (PMC11337648; doi:10.1186/s13071-024-06431-1)
Supplement: Supplementary file 5 — Additional file 5. Table S2: Promoter sequences used in this study. [file 13071_2024_6431_MOESM5_ESM.docx]

**Table S2.** Promote sequences used in this study

| Name | Sequence (5'→3') |
| --- | --- |
| pcgd1_1570 (p*E2F*) | TGGCTACTTTAATTTTTACTCAAATTTGAACTATCCTATAAAATCTTAAATTATTAGCCCTAACCGCTGTTCTCTAAATTGACCTGTGCAAAACGAATTTGCGAGGAGCGCCAGAAATAAGGCGCCTTGGTGCTTTTTGCGGTTAATGTAAATTTAGCCCCAATATAAGGTTTAGTAGCAATTAATTTGAGCAAATTTTTTACTTCAGAGAATAATTTTTTTGCAGTAAATAATATTGACTGCACTCATTGATTATATCGCTCAATTCTGATATAGTTTGTTTGGAAAGGCGAGGCCTACTTAATTGGTTTCTCATTTTAGGGAGAGGGGCTAAAAGAATTAGACAACAGTTTCTATTCCGAATTTAGCTGGTAAAA |
| pcgd2_1360 (p*ATPase*) | TGGTATTACAACGTAAATTAACTCAACTATTTGGAAAATTACTCTTGCAATAATATTTCCCCCCACCCACAGCAATTTGATTTTGTTTAACACCCGCTCTAAATCTGTTTAGTACTTAAGATAATTACATTCTATTTTGAATTTTAATTGTGATTCTCTTAACACTTTCATCTTACTCATTACAATTGTTCACTACTTATTCCTGTTTTGAAAATTTCTCTATTTTTGTATTGTTTTTATATTTAACTTCTACTATTTTGCCTTCTCGCTATTAAATATACTCAGTTCCTTATTAAACATTGGATATTGATTTTTAGAATATATTTCCTATTTTTATTCTCTTGTTTATTCGCAAAAATTAAATTGCATAGAATCCGGCTTTCATGCAATTTTGCACAATTACTTTTTAATTTTAAATTAAAGAGTGGAGGCA |
| pcgd1_2270 (p*40S ribosomal protein S26*) | GCAGAAATAACCTTCAAAATAAAGTTCGGTACCAACGTTTCAAGGCGCGTTAATGGAATAGTTTAGGCGCTAGAGACGGATTATATTATTTCATTTAACTGAATTTGTCTAATAAATATATTCATTGGAGACGTGAAATTTAGTTAAATTTTGAAGAAGTTCACTGTAAAACTTAAAGGGATTTTTTAGACTTTCGAGTATTTTAGATTGTCTTGAATCTTTTCTAATTGACTTTGAGTAACTGTTTTTTTAATTGTTAATTTGTTGACA |
| pcgd3_260 | ATAGTATCCAAACAATGAATTGTTTGACTACTTTTATCTGTTTTTATTGAAAATGAGTCTACCGCAAATATTCAGTTAGAAAATCTAACCCCTATCTAGAACTGTTCATGAAAATAAACATATTTCCATATTGCATGCAATAATTAAAATTCGCAAAAATATTACTTCCCAATAAAAATATATTCGCGCATGCAATAGCCACTATTGATAATTTACTTTAATTTTATTGGGCCTCCCCTTTTATCTCTCTATCATTGGCGCCTAGGCGGTACCGATTGAAAATGAATTCAAAGAAAATATACAACTAGAAATATAGATATCGTAAACAAAGTTTTTTGCCAAATTAAGCTATCTAAAGTATGCTTATGGGGAATAACTCAGTGTGTTAAGCATTTTTTTGAGAAATAACATGTATTTAAGTAGAAAAGAAGGAAAAG |

**Table S2.** The promote sequences used in this study (continued)

| Name | Sequence (5'→3') |
| --- | --- |
| pcgd4_2860 (p*α-Tubulin*) | ATAAAATTTAGCACACTAATTCAACTTTCCACTGAACGTTTTTTCATTCTTTTCGGTTACTTGAATTAATTCCCGCCTTATCCCACGTTTGGTTCGACCCGCAAATTAGAGTGGACAGCCCCCACAAATAAAACGTTAAGCATGCCTGTGAAAGAACTGCCATCAAAATCGTCTTTGTTCTCTGAAGCTTGACCTCCTCATTGGTTTCTTCTCAGGATACTCTTAATTATATAATACTCTTTGTTAAAGTTATTCGTTAAAC |
| pcgd5_1960 (p*Enolose*) | TGGGGAAACTAAATATACTGAAATTCGGTAGATTCTATATCTCACGGGACAGCTTTTCACACACACTTAGTTCTATATTGCGTCATAACTTTTGTTTTTTTTGTTGCACTTTTTTTCTCTTATATTCAAGTAAGTGGTTTAGATTCTCTAAGGGCGGGAATATGAATTAGTGGCAATTCAAAGGATTTAAACGATCAGGCGCCTGCACACCAAACTCTAATTCGTACACAGCATGCCCGAGGTTATAGATATAGATACTGCGAAATTATTTTCATTGTTTCAGTTAAGAAATAATAAAATATTTTATTATAGTTATTTTCCAAATTTATTTGAGATTTTTGTATTGAAGTTTAGCC |
| pcgd5_3160 (p*Actin*) | TCAGAATGAGTTGGTTATAAACAGTAATAATAGAAAGCGTTAGGGCTATAAAAATGACCAAATAAGTCATTTAGCATCATAAGTATATACATTAGATACAGAATGAGTTCAAAATAAATAAAAAATTAGACTTACTTACAAATTCTAAGCTATAGTATAAAAGAACTAAGAAGATTTGATGAATTTGCTATACCATTTTAAATATTTGTTTATAGTTTTTTGATATTAATATAAGGACTATTAAGTGAAAAGGAAACTACACATATTTCCGCCAATTCATTGTATTGCATCGCACACATAAATGAAAGATTAAAAGAAAAGAAATTCTATTATCTAGTATTAAAATAATACCAATAATAAGCAACACTTTTTTGTTATTTTTTATTATAAAGATTAATTTGCTACTTTAAATTGAAAAAAACTTTACAGGTTTATACAAATTCGTACATCACATGTATACAATGCTACATACATTACTTGATAAATATTACGACGTGAAACGTATTAATTTAAACGAATCTTAGTTATGTAAATAAAAAAAGGTTAGATTTTAGTAAAAAAAAAAGACCAAATTTTATATTAAATATAAATCTTGCATTGAATGCATTTATGTCAAATTGCATGCACCTTGCCGCCCACCATGCATAGACGCGCGCCAATACGTATTAATAGACGATTACATGACAAAGTTTAGAGATTAAATAAAAATCAAGTATAAATTTAGAATAGACAACATGTAAAAAGTGAGATGAATCTTTTTACGATACGTACAGTGCGCATTAAAAATCCAAAGTATATTTTTATTTTCCTGCGTGCGTATACATATCTTTAAATACTTGGATAAAGAGATAATAAGAATTGTACGGCAAGCAAAGTAGTAGTTTAATAGAGCAGTTTTTATATTTTATATATAGCTTACTTACAATATAGATTTTTTTTTTTCATAGATTTCGGGCATATTACACAAGTAACAAAAG |

**Table S2.** The promote sequences used in this study (continued)

| Name | Sequence (5'→3') |
| --- | --- |
| pcgd6_4110 | ATAAAGTGCATGGTAGCCCGCCTTTTTATCATCCCCAAGAGTCGTTCAAGCGAATTGTGAAAATCAAATAATGCAAAATAAAGATATCAATCAAGCATAAATGAAAACTAGTACATACCATTTTCATATTCTTACATTCATAAATATTCACATTTATGGGTTATTCACAATTTCACATACTTCTTTTTAATATATATTAATTTCAGATACAAATATTTAATGGGTATACGTACACGCACGTCTCCATTCAAAATGTCACCCCAAAAAAAAGTGTATGTAACTATCCGTCTGTTGTTGTCTCTTGGGGTTGGCGCAACGCACATACAATAGTCAAAATCTGAACAAGTAATAACTTTTGTGTATGGGAGTGGGCTAATTAGTGGATTATCAGGAATCTTATAAAA |
