# Supplementary material for: Optimization of a DiCre recombinase system with reduced leakage for conditional genome editing of Cryptosporidium
Source: Parasit Vectors. 2024 Aug 21;17:352. doi: 10.1186/s13071-024-06431-1 (PMC11337648; doi:10.1186/s13071-024-06431-1)
Supplement: Supplementary file 6 — Additional file 6. Table S3: Primers used in this study. [file 13071_2024_6431_MOESM6_ESM.docx]

**Table S3.** Primers used in this study

| Primer name | Sequence (5'→3') | Specific purpose |
| --- | --- | --- |
| StopLoxP-Nluc line-5' Insert-F | CGCTTTCAACAATAACTGGTTCA | For verifying the 5' correct gene integration of the StopLoxP-Nluc line |
| StopLoxP-Nluc line-5' Insert-R | TTAGCCCACTCCCATACACA |  |
| StopLoxP-Nluc line-3' Insert-F | GCTGAAGAACTTGGTGGTGA | For verifying the 3' correct gene integration of the StopLoxP-Nluc line |
| StopLoxP-Nluc line-3' Insert-R | TTGATGAAGAAGGAGGAATAAACC |  |
| TK-F | ATTAGCATGGGCTGATAAACTTACT | For amplifying the *TK* locus |
| TK-R | ATATGATGTTTTCTGCATACAGAAG |  |
| Control-F | ACTTTGAGTGGGAGGAAG | For amplifying the *INS-3* locus |
| Control-R | AGCGAGTCAGATACAAGC |  |
| Nluc-neoLoxP-mNG line-5' Insert-F | CGCTTTCAACAATAACTGGTTCA | For verifying the 5' correct gene integration of the Nluc-neoLoxP-mNG line |
| Nluc-neoLoxP-mNG line-5' Insert-R | TTAGCCCACTCCCATACACA |  |
| Nluc-neoLoxP-mNG line-3' Insert-F | GCTGAAGAACTTGGTGGTGA | For verifying the 3' correct gene integration of the Nluc-neoLoxP-mNG line |
| Nluc-neoLoxP-mNG line-3' Insert-R | TTGATGAAGAAGGAGGAATAAACC |  |
| Cre-F | TATTATTCCACCACATGCTACTT | For amplifying the *Cre* locus |
| Cre-R | ATCTCTAACATCTTCAGGCTCAG |  |
| Nluc-F | CAAGGCGCGTTAATGGAATAG | For amplifying the *Nluc* locus |
| Nluc-R | GGCAGGAGCAAGGTGAGAAGA |  |
| LoxP-F | CAAGGCGCGTTAATGGAATAG | For verifying target sequence excision between loxP sites in the genome of the StopLoxP-Nluc line |
| LoxP-R | ATGAGTAGCAGGTAATGAAGCCA |  |
